# Management of HIV-1 associated hepatitis in patients with acquired immunodeficiency syndrome: role of a successful control of viral replication

**DOI:** 10.1186/1742-6405-8-9

**Published:** 2011-03-01

**Authors:** Antonella Esposito, Valentina Conti, Maria Cagliuso, Daniele Pastori, Alessandra Fantauzzi, Ivano Mezzaroma

**Affiliations:** 1 Department of Clinical Medicine, "Sapienza"- University of Rome, Rome, Italy; 2 Department of Experimental Medicine, "Sapienza"- University of Rome, Rome, Italy

## Abstract

In HIV-1 infected patients, increase of liver enzymes may be mainly due to viral coinfections, alcohol intake, hepatotoxic drugs or autoimmune diseases. Three cases of aminotransferase elevation occurred during a phase of uncontrolled viral replication combined with a severe immunodeficiency and resolved by an effective HAART are described, focusing on the etio-pathogenetic role possibly played by HIV-1 infection.

## Background

Human immunodeficiency virus type-1 (HIV-1) infection is commonly characterized by the presence of a progressive depletion of CD4^+ ^T lymphocytes, associated with the occurrence of opportunistic infections and cancers. Abnormal liver enzymes (aspartate aminotransferase, AST and alanine aminotransferase, ALT) are frequently seen in HIV-1 infected patients and may be due to a variety of factors, such as coinfection with hepatotropic viruses, i.e. hepatitis B (HBV) and C (HCV) viruses, Cytomegalovirus (CMV) and Epstein-Barr virus (EBV), opportunistic infections, cancers, autoimmune hepatitis (AIH), alcohol abuse, and exposure to hepatotoxic drugs, including highly active antiretroviral therapy (HAART). Identification and management of these factors is often difficult because of the coexistence of multiple causes [1-4].

We report three cases of HIV-1 infected patients with advanced immunodeficiency (CD4^+ ^T lymphocytes less than 200/cu.mm.) showing severe aminotransferase elevations (defined as ≥ 5× the upper limit of normal values) occurred during a period of uncontrolled viral replication, and in the absence of any other apparent cause of liver disease. AST and ALT values returned within the normal ranges in a few months after the onset of an effective HAART.

## Case Presentation

### Case 1

**Figure 1:**
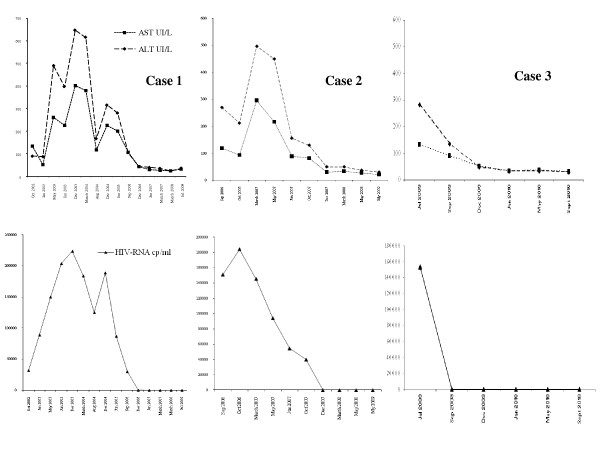
**Trend of HIV-1 RNA and ALT/AST levels**. Aminotransferase (AST and ALT) values and plasma HIV-RNA levels in the three patients before and after the beginning of an effective HAART. Normalization of both indexes after the start of the new combination therapy.

On January 1987, a 30-years-old man who have sex with men (MSM) was found to be HIV-1 positive (CDC A2) for a history of unprotected sexual intercourses. On September 1990 the patient has presented a CMV retinitis and a zidovudine-based antiretroviral therapy was started. From 1990 to 2002 he switched several antiretroviral treatments, due to side effects or development of drug resistance. During this period a partial immune recovery was observed and plasma HIV-RNA levels sometimes reached undetectable values, with liver function tests (LFTs) always within the normal ranges. On October 2003, increased levels of AST (135 UI/l) and ALT (89 UI/l) were present. Moreover, the patient showed a failure of the lopinavir/ritonavir-based therapy (HIV-RNA 32.000 copies/ml; CD4^+ ^T lymphocytes 95 cells/cu.mm.). Not excluding a drug toxicity, HAART was discontinued and the patient underwent to a complete assessment of hepatic functions, including HAV, HBV and HCV antibodies, HBV-DNA, HCV-RNA, EBV-DNA and CMV-DNA, all resulted negative. A genetic test for hemochromatosis and the research of auto-antibodies for AIH were also negative. No diabetes or other metabolic disorders, with the exception of a mild increase in triglycerides, were present. A liver ultrasound showed hepatomegaly without parenchymal abnormalities. A liver biopsy revealed hepatosteatosis with multifocal lymphocytic lobular infiltrates. From August 2004 to August 2006, despite the presence of a multi-drug resistant virus, antiretroviral therapy was reintroduced, with the intent to delay the clinical evolution of HIV-1 disease (Table 1). During these years an immune-virological worsening (CD4^+ ^T lymphocytes 36 cells/cu.mm.; HIV-RNA 223.805 copies/ml) and a deterioration of AST and ALT values (400 UI/l and 600 UI/l respectively) were observed. On August 2006, a darunavir/ritonavir + enfuvirtide + tenofovir and lamivudine/zidovudine fixed-dose regimen was started, based on the results of a genotypic resistance test (GRT) and the availability of new drugs. After one month, HIV-RNA levels decreased to 100 copies/ml and AST and ALT values returned below 50 UI/ml. Three months later, plasma HIV-RNA was undetectable whereas LFTs reached normal levels. After one year, enfuvirtide was replaced with raltegravir, and lamivudine/zidovudine fixed-dose + tenofovir discontinued. Plasma viral load has remained on undetectable levels and CD4^+ ^T lymphocytes have now reached 430 cells/cu.mm., with LFTs always within the normal ranges (Figure 1).

**Table 1:** Therapeutic history of the patients.

Antiretroviral drugs	Period of therapy	Reasons for change
**Case 1**		

ZDV	1990 -1993	

ddI	May 1993 - Oct 1993	diarrhoea

ddC	Dec 1993 - Jan 1995	

ZDV, 3TC	Feb 1995 - Jul 1995	

ZDV, 3TC, SQV	Aug 1995 - Feb 1996	diarrhoea

IDV, 3TC, ZDV	Mar 1996 - Apr 1998	kidney stones

d4T, EFV, NFV	May 1998 - Apr 1999	psichiatric disorders

ZDV, 3TC, ABC, IDV/r	May 1999 - Jan 2000	cutaneous reaction

ZDV, 3TC, IDV/SQV/r	Feb 2000 - Sep 2000	virologic failure

ddI, d4T, NVP, LPV/r	Oct 2000	cutaneous reaction

ddI, d4T, EFV, LPV/r	Nov 2000 - Oct 2001	

ddI, d4T, LPV/r	Nov 2001 - Oct 2003	virologic failure and LFTs increase

3TC, TDF, EFV	Aug 2004 - Jun 2005	failure

fAPV/r, SQV	Jul 2005	cutaneous reaction

SQV/r, EFV	Aug 2005 - Oct 2005	cutaneous reaction

LPV/r, SQV	Oct 2005 - Dec 2005	itching and diarrhoea

LPV/r, 3TC, TDF	Dec 2005 - Jun2006	failure

DRV/r, ENF, TDF, ZDV/3TC	Aug 2006 - Aug 2007	

DRV/r, RAL, TDF, ZDV/3TC	Aug 2007 - Aug 2008	simplification

DRV/r, RAL	Aug 2008 - ongoing	simplification

**Case 2**		

ZDV, ddI	Nov 1995 - Aug 1996	

ZDV, ddC	Sep 1996 - Dec 1996	

ZDV, ddC, IDV	Dec 1996 - May 1997	

ZDV, 3TC, IDV	Jun 1997 - Nov 1998	kidney stones

NFV, d4T, NVP	Dec 1998 - Mar 2000	abdominal pain and diarrhea

ZDV/3TC, SQV/r	Mar 2000 - Apr 2000	anemia

d4T, 3TC, SQV/r	May 2000 - Mar 2005	lack of adherence and resistance development

LPV/r, 3TC, TDF	Apr 2005 - Oct 2005	virologic failure

ATV/r, 3TC, TDF	Oct 2005 - Sep 2006	virologic failure and LFTs increase

DRV/r, ENF, RAL	Oct 2007 - Feb 2008	

DRV/r, RAL	Feb 2008 - ongoing	simplification

**Case 3**		

IDV, 3TC, d4T	Dec 1997 - Mar 2002	

NVP, 3TC, d4T	Apr 2002 - May 2004	simplification

NVP, 3TC, TDF	Jun 2004 - Apr 2006	peripheral neuropathy, lipoatrophy

RAL, TDF/FTC	Aug 2009 - ongoing	

Note: GRT, genotypic resistance test; ZDV, zidovudine; ddI, didanosine; ddC, zalcitabine; 3TC, lamivudine; SQV, saquinavir; IDV, indinavir; d4T, stavudine; EFV, efavirenz; NFV, nelfinavir; ABC, abacavir; NVP, nevirapine; LPV, lopinavir; TDF, tenofovir difumarate; fAPV, fosamprenavir; DRV, darunavir; ENF, enfuvirtide; RAL, raltegravir; ATV, atazanavir; FTC, emtricitabine; r = ritonavir booster.

### Case 2

A 31-years-old man on September 1995 was found to be HIV-1 positive during a hospitalization caused by *Pneumocystis jiroveci *pneumonia (CDC C3). He started antiretroviral therapy on November 1995 with zidovudine and didanosine, when his CD4^+ ^T cell count was 32 cells/cu.mm. He reported a history of unprotected heterosexual intercourses. Up to September 2006, he switched several treatments for side effects or for the occurrence of drug resistance (Table 1), caused by a low level of adherence to the prescribed therapies. Despite this, the patient showed an immune recovery with CD4^+ ^T cells increased up to 600/cu.mm: his LFTs were predominantly within the normal ranges, with only small increases due to occasional alcohol intake. On September 2006, during a rescue treatment with atazanavir/ritonavir, lamivudine and tenofovir, the patients experienced a new virologic failure (HIV-RNA 150.803 copies/ml) with a worse in CD4^+ ^T cell count (55 cells/cu.mm.) and an increase of LFTs (ALT 119 UI/l and AST 270 UI/l). No diabetes or other metabolic disorders were present. Antiretrovirals were discontinued and the patient underwent to laboratory and instrumental examinations to investigate the aetiology of the liver disease: HBV, HCV, CMV and EBV antibodies, and the relative DNA or RNA detections by polymerase chain reaction (PCR), were negative as well as the auto-antibody titres. A liver ultrasound showed only a mild hepatomegaly, whereas a liver biopsy revealed mild hepatosteatosis without fibrosis, and focal lymphocytic lobular infiltrates. The patient remained without antiretroviral therapy until October 2007, when, despite the persistence of abnormal LFTs, the treatment was restarted with a darunavir/ritonavir + enfuvirtide + raltegravir-based HAART, taking in account the results of a new GRT. After only one month of therapy, LFTs returned within the normal ranges and two months later plasma HIV-RNA values reached undetectable levels (Figure 1), both in association with a sustained immune recovery (CD4^+ ^T lymphocytes raised from 15 to 211 cells/cu.mm.). Actually the patient is taking a simplified regimen with darunavir/ritonavir + raltegravir, his CD4^+ ^T cells have reached 359/cu.mm., with plasma HIV RNA always below the limits of detection and LFTs within the normal ranges.

### Case 3

The third patient is a 47-years-old MSM, HIV-1 positive from December 1997, when the research of anti-HIV-1 antibodies was performed for the onset of disseminated Kaposi's sarcoma (KS) lesions with visceral involvement (CDC C3). HAART with indinavir, lamivudine and stavudine was started, shortly allowing an immune-virological recovery. After three months of therapy, CD4^+ ^T lymphocytes raised from 155 to 461 cells/cu.mm. and plasma HIV RNA decreased from 251.000 copies/ml to undetectable levels. KS lesions disappeared and an amelioration of clinical conditions was observed. Up to April 2006, he continued HAART, switching from indinavir to nevirapine for simplification, and from stavudine to tenofovir for the onset of peripheral neuropathy. During this period plasma HIV RNA remained undetectable and CD4^+ ^T lymphocytes were constantly over 500 cells/cu.mm. LFTs were always within the normal ranges or showed little abnormalities, clearly referred to the use of specific antiretroviral drugs (hyperbilirubinemia and gamma-glutamil-transpeptidase mild increases with indinavir and nevirapine, respectively). HBV and HCV serology were negative, whereas anti-CMV and anti-EBV IgG antibodies were present. From April 2006 the patient was no longer subjected to clinical and laboratory scheduled follow-up. He discontinued HAART for the onset of lipodistrophy signs, and on July 2009 he returned to our clinic, showing a worsening of clinical conditions (fever, weight loss, night sweats, and oropharyngeal candidiasis). Laboratory tests revealed: HIV-RNA 153.465 copies/ml, CD4^+ ^T lymphocytes 222 cells/cu.mm., ALT 131 UI/l and AST 282 UI/l. HBV and HCV serology remained negative as well as the relative DNA and RNA by PCR analysis; a liver ultrasound did not show signs of liver damage. Autoantibody research for AIH was negative. After the results of a resistance test, a new antiretroviral combination based on raltegravir plus tenofovir/emtricitabine fixed-dose was prescribed. A rapid amelioration of the clinical conditions was observed and after one month of therapy he showed an immune-virological recovery with a persistently improvement of LFTs (Figure 1).

## Conclusions

Involvement of the liver during the course of HIV-1 disease may result from viral or other infections, or being secondary to cancers, toxic agents and drugs. The three patients described here showed an advanced HIV-1 disease with an unexpected increase of LFTs occurred in the presence of a severe immunodeficiency (CD4^+ ^T cells < 200/cu.mm.) and a high level of viral replication, due to drug failure or voluntary HAART discontinuation. All patients showed plasma HIV RNA values > 100.000 copies/ml and the absence of other possible causes of liver injury, such as hepatotropic virus infections, hepatotoxic drugs administration, alcohol intake and/or other substances' abuse, or cancers. Furthermore, the search for other pathogens (i.e., typical or atypical mycobacterial infection, *Treponema pallidum *infection) resulted negative. Hepatic biopsy performed in two subjects was not of diagnostic value. A triggering role in the aminotransferase increase played by antiretroviral drugs (i.e. LPV/r and ATZ/r) at least in the first two subjects could not be excluded, being hepatotoxicity of these drugs frequently reported [5-7]. In all subjects LFTs rapidly returned within the normal ranges, as soon as the control of viral replication has been achieved; this was observed in association with an immune recovery, suggesting the hypothesis of a pathogenetic role played by HIV-1 infection in the liver damage. In patients with HIV-1 disease there are a few data on liver injury not related to hepatotropic viruses or hepatotoxic drugs [8-10]. An acute liver disease without the identification of a distinct pathogen can complicate the clinical evolution of HIV-1 infection in children [11]. Only a few cases of AIH, in which a role of HIV-1 as a causative agent was hypothesized, have been described [12,13]. Viral infections may play a triggering role in the activation of auto-reactive T cells that attack hepatocytes either for a molecular mimicry between viral and self-antigens or by the modifications of self-antigens; alternatively, HIV-1 may cause a superantigen stimulation of a subset of T cells responsible for the liver damage [13]. However, in our patients the detection of auto-antibodies was negative, although their absence does not allow us to absolutely exclude the diagnosis of AIH, and the liver biopsy was not diagnostic. Hepatosteatosis was a common autopsy finding in HIV/AIDS patients, extensively described in the pre-HAART era [8]. In the LFTs increase observed, a role of steatosis, whose presence was clearly demonstrated in the liver biopsies performed in two patients, could not be excluded. However, in our series the aminotransferase increase has promptly resolved after the start of an effective HAART, without interventions finalized to reduce hepatosteatosis. Indeed, as previously reported [14], such approaches lead to an improvement of antiretroviral drug tolerability only in coinfected patients.

In conclusion, in HIV-1 infected patients presenting with an acute onset of abnormal LFTs, after the exclusion of the most common causes of liver disease, it is necessary to assess the effectiveness of the current HAART and strongly evaluate the opportunity of starting an alternative antiretroviral regimen, in the suspicion of a HIV-1 induced hepatic disease.

## Consent

Written informed consent was obtained from the patients for publication of this case report and accompanying images. A copy of the written consent is available for review by the Editor-in-Chief of this journal.

## Competing interests

The authors declare that they have no competing interests.

## Authors' contributions

All authors read and approved the final manuscript, and significantly contributed to the work.
